# Age-related cardiopathies gene editing

**DOI:** 10.18632/aging.101853

**Published:** 2019-03-10

**Authors:** Moshi Song, Juan Carlos Izpisua Belmonte, Guang-Hui Liu

**Affiliations:** 1 State Key Laboratory of Membrane Biology, Institute of Zoology, Chinese Academy of Sciences, Beijing 100101, China; 2 Institute for Stem Cell and Regeneration, CAS, Beijing 100101, China; 3 University of Chinese Academy of Sciences, Beijing 100049, China; 4 Gene Expression Laboratory, Salk Institute for Biological Studies, La Jolla 92037, USA; 5 National Laboratory of Biomacromolecules, CAS Center for Excellence in Biomacromolecules, Institute of Biophysics, Chinese Academy of Sciences, Beijing 100101, China; 6 National Clinical Research Center for Geriatric Disorders, Advanced Innovation Center for Human Brain Protection, Xuanwu Hospital Capital Medical University, Beijing 100053, China

**Keywords:** CRISPR/Cas9, gene editing, aging, cardiac diseases, cardiopathy

Aging is associated with progressive functional and structural deterioration of the heart and is a dominant risk factor for cardiac diseases, which are the leading causes of death worldwide. It is predicted that by the year 2035, a quarter of the population worldwide will be over 65 years old. Due to the increasingly growing elderly population, there is an urgent need for developing novel interventions against and understanding the molecular mechanisms of age-related cardiac diseases [1].

Genome editing is referred to as technologies that add, remove, or alter genetic materials at particular locations in an organism’s genome. During the last decades, multiple approaches (including traditional meganucleases, ZFNs, TALENs, CRISPR/Cas9) have been established. To date, CRISPR/Cas9, short for clustered regularly interspaced short palindromic repeats and CRISPR-associated protein 9, is the most popular tool for genome editing due to its faster, cheaper, more accurate editing ability compared to other existing methods, though, as pointed out by Hernandez-Benitez et al [2], technical limitations of this technology, such as low efficiency, mosaicism, and off-target effects, remain to be overcome in combating cardiac aging.

**Figure 1:**
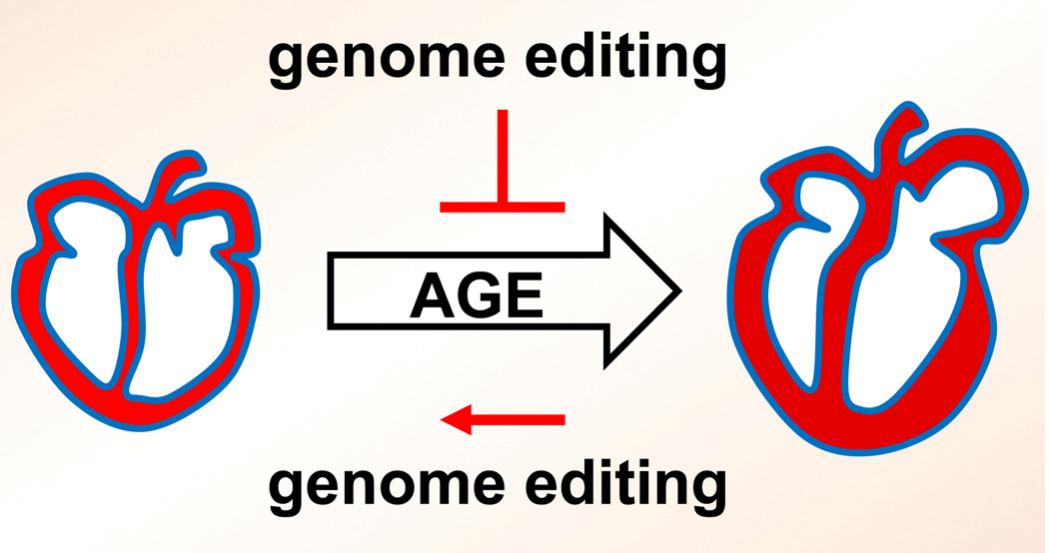
Schematics indicating regulation of cardiac aging by genome editing.

Intrinsic cardiac aging is accompanied by an increased prevalence of diastolic dysfunction, left ventricular (LV) hypertrophy, atrial fibrillation, and so forth, independent of common extrinsic risk factors [3] (Figure 1). Phenotypes of cardiac aging having been well-characterized but less is understood about the underlying molecular mechanisms. A number of events including calcium dyshomeostasis, miRNA deregulation, mitochondrial dysfunction and reactive oxygen species (ROS), and adverse extracellular matrix remodeling occur during cardiac aging, though the causative links between the key molecules involved in these abnormalities and cardiac aging (a chicken and egg situation) are mostly, as of yet, unclear [4]. It is important to first identify the key causative genes during cardiac aging and elucidate their exact roles under various circumstances before CRISPR/Cas9 could be applied. In addition, changes in the abundance of gene transcripts such as αMHC and SERCA2 are often observed in and likely contribute to cardiac aging. In this regard, Hernandez-Benitez et al [2] believe that CRISPR/Cas9-mediated transepigenetic regulation is of great potential to modulate gene expression via the targeted recruitment of transcriptional activation complexes [5].

One of the biggest limitations of CRISPR/Cas9-mediated genome editing in combating cardiac aging is that adult cardiomyocytes do not often divide, whereas CRISPR/Cas9 is most effective when cells do. As summarized by Hernandez-Benitez et al [2], the past attempts by inducing postmitotic cells to divide failed to improve the therapeutic effects of CRISPR/Cas9-mediated genome editing as the resulting differentiated cells are functionally compromised [6,7]. Yet, it was recently reported that the contemporaneous expression of cell-cycle regulators CDK1, CCNB, CDK4, and CCND leads to extensively increased proliferation of adult cardiomyocytes and significantly improved cardiac function following myocardial infarction, offering a potential approach to overcome this limitation [8].

As emphasized by Hernandez-Benitez et al [2], genome editing has greatly broadened our view of the mechanistic events taking place during cardiovascular diseases and in certain cases enabled correction of gene mutations during development. However, at this time, the success from CRISPR/Cas9-mediated or any other existing method of genome editing in combating cardiac aging in clinical settings is still beyond reach due to our lack of comprehensive understanding of the molecular events during cardiac aging and complete control of the editing process. We hope that continuous efforts by the scientific community will lead to new molecular interpretations of cardiac aging and even more delicate and efficient tools for genome editing, and potentially offer therapeutic strategies against human cardiac aging.
